# Cauda Equina Syndrome in a Military Personnel: A Case Report

**DOI:** 10.31729/jnma.8173

**Published:** 2023-06-30

**Authors:** Anup Rai, Kiran Ghimire, Omkar K C, Samir Pudasaini, Sumnima Rai

**Affiliations:** 1 Nepalese Army Institute of Health Sciences, Sanobharyang, Kathmandu, Nepal

**Keywords:** *case reports*, *cauda equina syndrome*, *disc herniation*, *low back pain*, *military personnel*

## Abstract

Cauda equina syndrome is a rare but severe neuro-spinal disorder commonly caused due to lumbar disc herniation, which occurs mostly at lower levels of L4-S1. We report a case of 38-year-old male soldier deployed on a foreign mission who presented to a level 1 military hospital 4 months back with complaints of decreased movement of bilateral lower limbs and severe low back pain radiating to the right lower limb for 2 hours. He was referred to a higher centre and diagnosed with cauda equina syndrome due to massive disc herniation at levels L2-L3. He underwent laminotomy and discectomy of the extruded intervertebral disc after 48 hours. On subsequent follow-up, his bladder and lower limbs sensations were normal however, he had bowel incontinence, hypotonia, hyporeflexia, and no significant improvement in power. Hence, early diagnosis, referral, and timely intervention affect the outcomes in a cauda equina syndrome patient.

## INTRODUCTION

Cauda equina syndrome (CES) has a crude incidence of 2.7 per 100,000 person-year in the general population and 7 per 100,000 person-year in the military.^1,2^ It is commonly caused by lumbar disc herniation (LDH), followed by tumours, infection, and stenosis.^3-5^ Most LDH occurs at the L4-S1 level, which requires immediate surgical intervention. But even after timely surgical management, long-term morbidity and outcomes can vary. The military has many risk factors, like a history of lower back pain (LBP), past musculoskeletal injuries, female sex, and lower rank.^6^ Here, we present a case of CES in a young soldier secondary to massive disc herniation at level L2-L3.

## CASE REPORT

A 38-year-old male soldier presented to level 1 military hospital 4 months back, with complaints of decreased movement of bilateral lower limbs and severe low back pain radiating to the right lower limb for 2 hours. He also complained of difficulty initiating urination and was unable to defecate. He had no significant past medical or surgical history and was certified medically fit for deployment in a foreign mission.

On examination, Lasegue's test was positive on both limbs. The modified medical research council (MMRC) grade of muscle power for bilateral lower limbs was 4/5 on flexors and extensors of the thigh, 3/5 in the ankle, and 3/5 in the extensor/flexor hallucis longus (EHL/ FHL). The tone of bilateral lower limbs was reduced. The right lower limb's L5/S1 dermatome showed decreased fine touch sensation. Babinski's sign was mute bilaterally, along with diminished patellar reflexes. However, crude touch, pressure sensation in the limbs, and perineal sensation were intact.

On regular examination after 24 hours, his bilateral lower limb power was 0/5 with hypotonia, hyporeflexia, and 2/2 sensation in the L1-S1 region. The anal tone was normal on digital rectal examination (DRE).

**Figure 1:**
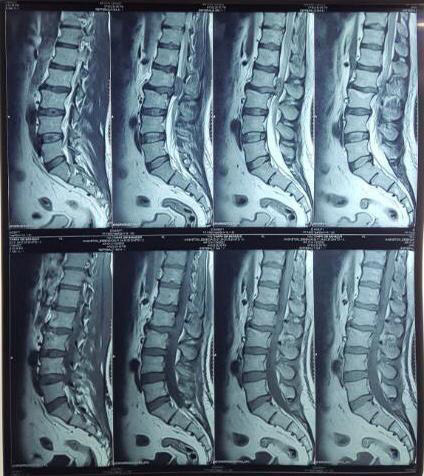
T2 sagittal section MRI of Lumbo Sacral Spine.

Within 24 hours of the onset of symptoms, medical evacuation to a higher centre with the facility of advanced medical imaging and neurosurgical care was done. A lumbosacral X-ray showed degenerative changes in the L2-L3 region. The lumbosacral spine magnetic imaging resonance (MRI) with whole-spine screening revealed a large disc extrusion at L2/L3, measuring 2.3x1.2 cm and migrating superiorly within the spinal canal, as well as significant central stenosis with compression of the thecal sac and cauda equina (Figure 1).

**Figure 2:**
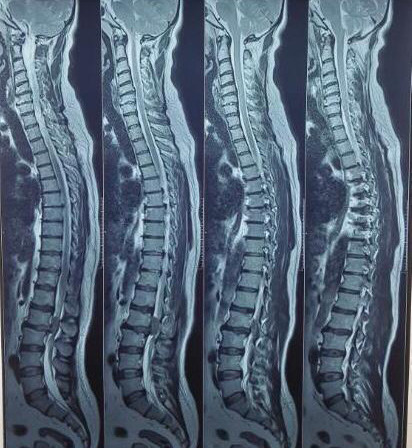
T2 sagittal section MRI of the cervical and thoracic spine.

There was a minimal posterior disc bulge at L3/L4 with no nerve root compression. There were mild posterior bulges but no cord or nerve root compression in the C5/C6/C7 region (Figure 2).

A routine blood investigation showed leucocytosis with neutrophil predominance. However other blood investigations were within normal limits.

Initially, the patient was managed with a dose of intramuscular injection of diclofenac 75 mg, intravenous (IV) corticosteroid injection of 120 mg, and injection of morphine as needed. After 48 hours, he underwent laminotomy (L2-L3) and discectomy of the extruded intervertebral disc.

On the second postoperative day, the patient was stable and anal tone was normal on DRE. He was started on postoperative analgesics, IV antibiotics, SC anticoagulants, physiotherapy, and bladder training exercises. On the sixth postoperative day, he developed muscle spasms in the right lower limbs, and muscle relaxants were prescribed to him. On arrival at Shree Birendra Hospital, Kathmandu after a month, he was admitted for physiotherapy and rehabilitation. His power on the left lower limb was 0/5 and 1/5 on the right side with hypotonia, hyporeflexia, and intact touch sensation. The bilateral perineal sensation was 0/2. On subsequent follow-up after 3 months, lower limb sensation was normal with a power of 2/5 on the right lower limb, and on the left side, there was fasciculation with a power of 1/5 grade. The bladder sensation was present but he still had bowel incontinence.

## DISCUSSION

Amongst all disc herniations, less than 5% are upper lumbar disc herniation (T12-L4 intervertebral levels), and L1-L2 and L2-L3 discs comprise about 1 to 2% of them.^7-9^ The distinctive features of upper LDH are ill-defined polyradiculopathies that are difficult to classify as specific muscle group weakness, dermatomal sensory deficits, or reflex deficits. The rate of autonomic (bowel/bladder sphincter) dysfunction from a cauda equina lesion is as high as 27% with upper lumbar discs.^9^ Cauda equina syndrome can present in different stages with various features.^3^ Our case, presented in the CES suspected stage with features of moderate low back pain radiating more towards the right lower limb which progressed to the CES incomplete stage with complaints of difficulty in initiating micturition. There was no bladder incontinence as seen in the CES retention stage. After 24 hours, paraparesis a feature of the CES complete stage was seen but the perineal sensation was intact, anal tone was normal with no bowel or bladder incontinence.

In cases of upper LDH, selecting a definite surgical approach is always challenging due to its unique anatomy, low incidence, and delay in diagnosis. The surgical outcomes on the upper LDH are also less favourable than those on the lower LDH.^9^ In general, on long-term follow-up after surgery, nearly 75% of patients have bladder problems, and 40% have sexual dysfunction.^10^ There are recommendations that surgical intervention be done as early as possible, within 24-48 hours of symptoms. Those who received decompression within 48 hours showed a significant improvement in sensory and motor impairments, as well as urine and rectal function, compared to those after 48 hours. But still, there is debate concerning the precise timing of surgical intervention.^8^ In our case, laminotomy (L2-L3) and discectomy of the extruded intervertebral disc (IVD) were done 48 hours after the initial presentation. On follow-up after 3 months his lower limb sensation was normal. Power on the right lower limb was MMRC grade 2/5 while on the left side was 1/5 grade. The bladder sensation was present but he had bowel incontinence, thus showing some signs of recovery.

CES due to upper LDH occurs rarely and it differs in clinical presentation and outcomes after surgical management which we have presented here. In the case study, early recognition and referral to a higher centre for management represent the strength. However, the inability to regular long-term follow-up of the case for clinical assessment is the main limitation.

In conclusion, LBP is a common musculoskeletal condition among military personnel. Most of the time, it is not associated with clinically significant pathology, and there is always a possibility of conditions like CES getting overlooked. Therefore, while evaluating patients with LBP, doctors must take a relevant history, and perform a detailed physical examination, imaging tests, and consultations. In order to reduce morbidity and improve the quality of life in patients with CES, prompt access to medical care, early diagnosis, immediate evacuation, and timely intervention are necessary.

## References

[1] Woodfield J, Lammy S, Jamjoom AAB, Fadelalla MAG, Copley PC, Arora M (2022). Demographics of cauda equina syndrome: a population-based incidence study.. Neuroepidemiology..

[2] Schoenfeld AJ, Bader JO (2012). Cauda equina syndrome: an analysis of incidence rates and risk factors among a closed North American military population.. Clin Neurol Neurosurg..

[3] Long B, Koyfman A, Gottlieb M (2020). Evaluation and management of cauda equina syndrome in the emergency department.. Am J Emerg Med..

[4] Zhang J, Zhao F, Wang FL, Yang YF, Zhang C, Cao Y (2016). Identification of lumbar disc disease hallmarks: a large cross-sectional study.. SpringerPlus..

[5] McNamee J, Flynn P, O'Leary S, Love M, Kelly B (2013). Imaging in cauda equina syndrome--a pictorial review.. Ulster Med J..

[6] To D, Rezai M, Murnaghan K, Cancelliere C (2021). Risk factors for low back pain in active military personnel: a systematic review.. Chiropr Man Ther..

[7] N Ho DPE (2003). A case study of cauda equina syndrome.. Perm J..

[8] Ahn UM, Ahn NU, Buchowski JM, Garrett ES, Sieber AN, Kostuik JP (2000). Cauda equina syndrome secondary to lumbar disc herniation: a meta-analysis of surgical outcomes.. Spine (Phila Pa 1976)..

[9] Kim DS, Lee JK, Jang JW, Ko BS, Lee JH, Kim SH (2010). Clinical features and treatments of upper lumbar disc herniations.. J Korean Neurosurg Soc..

[10] Hazelwood JE, Hoeritzauer I, Pronin S, Demetriades AK (2019). An assessment of patient-reported long-term outcomes following surgery for cauda equina syndrome.. Acta Neurochir (Wien)..

